# Bayesian Rician Regression for Neuroimaging

**DOI:** 10.3389/fnins.2017.00586

**Published:** 2017-10-20

**Authors:** Bertil Wegmann, Anders Eklund, Mattias Villani

**Affiliations:** ^1^Division of Statistics and Machine Learning, Department of Computer and Information Science, Linköping University, Linköping, Sweden; ^2^Division of Medical Informatics, Department of Biomedical Engineering, Linköping University, Linköping, Sweden; ^3^Center for Medical Image Science and Visualization, Linköping University, Linköping, Sweden

**Keywords:** DTI, diffusion, fMRI, fractional anisotropy, mean diffusivity, MCMC, Rician

## Abstract

It is well-known that data from diffusion weighted imaging (DWI) follow the Rician distribution. The Rician distribution is also relevant for functional magnetic resonance imaging (fMRI) data obtained at high temporal or spatial resolution. We propose a general regression model for non-central χ (NC-χ) distributed data, with the heteroscedastic Rician regression model as a prominent special case. The model allows both parameters in the Rician distribution to be linked to explanatory variables, with the relevant variables chosen by Bayesian variable selection. A highly efficient Markov chain Monte Carlo (MCMC) algorithm is proposed to capture full model uncertainty by simulating from the joint posterior distribution of all model parameters and the binary variable selection indicators. Simulated regression data is used to demonstrate that the Rician model is able to detect the signal much more accurately than the traditionally used Gaussian model at low signal-to-noise ratios. Using a diffusion dataset from the Human Connectome Project, it is also shown that the commonly used approximate Gaussian noise model underestimates the mean diffusivity (MD) and the fractional anisotropy (FA) in the single-diffusion tensor model compared to the Rician model.

## 1. Introduction

Gaussian statistical models are very common in the field of neuroimaging, as they enable simple algorithms for estimation of brain activity and connectivity. However, the measured signal in diffusion weighted imaging (DWI) and functional magnetic resonance imaging (fMRI) is the magnitude of a complex-valued Gaussian signal and therefore follows a Rician distribution, see Gudbjartsson and Patz (1995) and section 2.1. The Gaussian model is a good approximation to the Rician model in fMRI as the signal-to-noise (SNR), defined here as the ratio of the average BOLD signal to its standard deviation, for fMRI data tends to be large enough for the approximation to be accurate (Adrian et al., 2013). However, the recent push toward higher temporal and spatial resolution in neuroimaging (Moeller et al., 2010; Feinberg and Yacoub, 2012; Setsompop et al., 2013) may lead to low SNRs with increased risk of distorted conclusions about brain activity and connectivity. Low SNRs are also common for DWI, especially when the *b*-value is high (Zhu et al., 2009). Using a Gaussian model for diffusion tensor imaging (DTI) can therefore lead to severely misleading inferences. The reason for the popularity of the Gaussian approach is that Gaussian models can be analyzed using simple algorithms, while the Rician distribution is complicated since it does not belong to the exponential family. More generally, MR images collected by simultaneous acquisition from *L* independent coils may follow the non-central χ (NC-χ) distribution with *L* degrees of freedom, depending on how the measurements are combined into a single image (Tristán-Vega et al., 2012; Aja-Fernandez and Vegas-Sanchez-Ferrero, 2016). We therefore derive our algorithm for the general NC-χ model from which the Rician model can be directly obtained as the special case when *L* = 1.

### 1.1. Rician models in neuroimaging

Rician models have mainly been used for noise removal in DWI (Basu et al., 2006; Awate and Whitaker, 2007; Aja-Fernandez et al., 2008; Wiest-Daesslé et al., 2008; He and Greenshields, 2009; Luisier et al., 2012). The common theme for these methods is to apply a Rician denoising filter, to restore the images to have no or reduced Rician noise, which often leads to simple and fast estimation of the restored images. However, this approach does not account for the uncertainty in denoising. This is in contrast to our approach where we explicitly model the Rician noise, thereby capturing the full uncertainty in the noise. Rician denoising models have also been used for HARDI (Descoteaux et al., 2008; Gupta and Awate, 2017), which uses a larger number of diffusion-weighting gradient directions and potentially larger *b*-values to obtain a more detailed restoration of the diffusion images.

There are also some examples of Rician models with explicit modeling of the Rician noise for tensor estimation (Andersson, 2008; Veraart et al., 2011). Although, these approaches use sophisticated techniques for estimating the diffusion tensors, they do not capture the full uncertainty of the model parameters in contrast to our Bayesian approach. In addition, the only method that we are aware of for estimating diffusion tensor parameters in the more general NC-χ regression model, for data acquired with several independent coils, is the (non-Bayesian) least squares approach presented in Tristán-Vega et al. (2012).

There have also been a handful of approaches for the Rician model in fMRI, but all of these have shown that the Gaussian model is a good approximation of the Rician model due to sufficiently large SNR levels (Solo and Noh, 2007; Zhu et al., 2009; Adrian et al., 2013).

### 1.2. Non-central chi and Rician regression

We introduce a NC-χ regression model where both parameters in the distribution (the mean and variance of the underlying complex-valued signal) are modeled as functions of variables, with the Rician model as an important special case. Our framework is more general compared to other approaches, as it is also possible to include variables to model the variance and not only the mean of the noise. We propose a Bayesian analysis of the model based on a highly efficient Markov Chain Monte Carlo (MCMC) algorithm, to simulate from the joint posterior distribution of all model parameters. The MCMC convergence is excellent due to an accurately tailored proposal distribution; see sections 3.4 and 4.5. A high efficiency makes it possible to use a smaller number of simulations to obtain the same numerical accuracy. This is absolutely crucial for imaging applications since a separate MCMC chain is run for each voxel. Moreover, our MCMC algorithm also performs Bayesian variable selection among both sets of variables. A Bayesian approach using MCMC has the obvious advantage of capturing the full uncertainty of the model parameters in each voxel. Andersson (2008) develops a sophisticated maximum a posteriori (MAP) estimation for the DTI model, but does not deal with posterior uncertainty. The posterior uncertainty, represented by the MCMC samples, can easily be propagated to the group analysis, to down-weight subjects with a higher uncertainty. This is in contrast to the popular TBSS approach (tract-based spatial statistics) (Smith et al., 2006) for voxel-wise multi-subject analysis of fractional anisotropy (FA), which ignores the uncertainty of the FA.

### 1.3. Results

A simulation study in Appendix B in Supplementary Material shows that our Rician regression model is remarkably adept at recovering the signal even at very low SNRs. We also show by visualizing brain activity within an fMRI context that the Gaussian model is likely to lead to severely erroneous inference in low SNR settings.

Using a freely available DWI dataset from the Human Connectome Project (Essen et al., 2013), we show that commonly used Gaussian DTI approximation underestimates the mean diffusivity (MD) and substantially underestimates the FA of the single-diffusion tensors, compared to the Rician model, especially in white-matter regions with high FA. In addition, we demonstrate that variables are needed in both parameters of the Rician distribution, not only in the mean.

## 2. Heteroscedastic Rician and NC-χ regression

We start by describing our model for the special case of a Rician distribution, and then generalize it to the NC-χ case.

### 2.1. Rician regression

The measured MR signal is complex-valued

$${ỹ}_{i}=a_{i}+b_{i}\cdot j,\text{ for }i=1,\ldots ,n,$$

where *j* denotes the imaginary number. The real part *a*_*i*_ ~ *N*(μ_*i*_ cos θ_*i*_, ϕ_*i*_) and the imaginary part *b*_*i*_ ~ *N*(μ_*i*_ sin θ_*i*_, ϕ_*i*_) are independent, and

$$\ln\mu_{i}=\beta_{0}+{\text{x}}_{i}^{\prime }\beta$$

is a linear function of a vector of variables **x**_*i*_ at measurement *i*. In fMRI the vector **x**_*i*_ typically contains the stimulus of the experiment convolved with a hemodynamic response function, polynomial time trends, and head motion parameters, while **x**_*i*_ mainly contains gradient directions in DTI. Note that ϕ_*i*_ is potentially measurement-varying, to allow for heteroscedastic complex-valued noise.

It is rare to analyze the complex signal measurements *a*_*i*_ and *b*_*i*_ directly (Rowe and Logan, 2004 and follow-up papers are exceptions). The most common approach is to use the magnitude of ỹ_*i*_ as response variable, i.e.,

$$y_{i}=|{ỹ}_{i}|=\sqrt{a_{i}^{2}+b_{i}^{2}}.$$

It is well-known that the magnitude follows a Rician distribution (Rice, 1945) with density function

$$p\left(y|\mu ,\phi \right)=\frac{y}{\phi }\exp\left(-\frac{\left(y^{2}+\mu^{2}\right)}{2\phi }\right)I_{0}\left(\frac{y\mu }{\phi }\right),$$

for *y* > 0 and zero otherwise, where *I*_0_(·) is the modified Bessel function of the first kind of order 0.

We propose the following heteroscedastic Rician regression model

(1) $$\begin{array}{ll} y_{i}|{\text{x}}_{i},{\text{z}}_{i} & \sim \text{Rice}\left(\mu_{i},\phi_{i}\right)\text{ for }i=1,\ldots ,n,\\ \ln\mu_{i} & =\beta_{0}+{\text{x}}_{i}^{T}\beta ,\\ \ln\phi_{i} & =\alpha_{0}+{\text{z}}_{i}^{T}\alpha , \end{array}$$

and independence of the *y*_*i*_ *conditional* on the variables in **x**_*i*_ and **z**_*i*_. Since **x**_*i*_ and **z**_*i*_ may contain lags of the response variables, our model can capture temporal dependence in fMRI. Note also that we allow for heteroscedasticity in the complex signal, since the variance of the underlying complex-valued signal ϕ_*i*_ is a function of the regressors in **z**_*i*_. Although, the model in Equation (1) has the same structure as a generalized linear model (GLM) (McCullagh and Nelder, 1989), it is actually outside the GLM class since the Rician distribution does not belong to the exponential family. The logarithmic link functions used in Equation (1) can be replaced by any twice-differentiable invertible link function.

It is generally agreed in the neuroimaging literature that the Rician model is a realistic description of the noise (Gudbjartsson and Patz, 1995; Lindquist, 2008), with the Gaussian model being a convenient approximate model at sufficiently high SNRs (typically above 3). We will therefore assume in the current paper that the Rician model is correct, and evaluate the difference between the Rician and Gaussian models.

### 2.2. NC-χ regression

Both fMRI and DWI images may be obtained from parallel acquisition protocols with multiple coils, often used to increase the temporal and spatial resolution. Under the assumption of independent complex Gaussian distributed noise in each coil, the sum of squared magnitudes follow the non-central χ (NC-χ) distribution (Tristán-Vega et al., 2012; Aja-Fernandez and Vegas-Sanchez-Ferrero, 2016). The non-central χ density with 2*L* degrees of freedom is of the form

(2) $$\begin{array}{l} p\left(y|\mu ,\phi ,L\right)=\frac{y^{L}}{\phi \mu^{L-1}}\exp\left(-\frac{y^{2}+\mu^{2}}{2\phi }\right)I_{L-1}\left(\frac{y\mu }{\phi }\right), \end{array}$$

for *y*, μ, ϕ > 0, where *I*_*L*−1_(·) is the modified Bessel function of the first kind of order *L* − 1. We denote this as *y* ~ NC-χ. Note that when *L* = 1, the density in Equation (2) reduces to the Rice(μ, ϕ) density. Similarly to the Rician case, we can model μ and ϕ as functions of explanatory variables via logarithmic link functions. In summary, the observations are assumed to be independently NC-χ distributed conditional on the explanatory variables, according to

(3) $$\begin{array}{ll} y_{i}|{\text{x}}_{i},{\text{z}}_{i} & \sim \text{NC}-\chi \left(\mu_{i},\phi_{i},L\right)\\ \ln\mu_{i} & =\beta_{0}+{\text{x}}_{i}^{T}\beta ,\\ \ln\phi_{i} & =\alpha_{0}+{\text{z}}_{i}^{T}\alpha . \end{array}$$

Lagged response values may again be used as variables in μ and ϕ to induce temporal dependence.

The order *L* of the NC-χ distribution may be given by the problem at hand, for example by the number of independent coils used for data collection. Due to the lack of perfect independence between coils and other imperfections, *L* is often unknown and needs to be estimated from the data. Note that *L* can in general be any positive real number in the NC-χ distribution, and does not need to be an integer. Our approach makes it straightforward to introduce an MCMC updating step, to simulate from the conditional posterior distribution of ln *L*, or even model ln *L* as a linear function of variables.

## 3. Bayesian inference

The Bayesian approach formulates a prior distribution for all model parameters, and then updates this prior distribution with observed data through the likelihood function to a posterior distribution.

### 3.1. Posterior distribution and posterior probability maps

The aim of a Bayesian analysis is the joint posterior distribution of all model parameters

p(β,α|y,X,Z)∝p(y|β,α,X,Z)p(β,α),

where *p*(**y**|β, α, **X**, **Z**) is the likelihood function for the MR signal, *p*(β, α) is the prior, $\text{y}={\left(y_{i}\right)}_{i=1}^{n}$, $\text{X}={\left({\text{x}}_{i}^{T}\right)}_{i=1}^{n}$ and $\text{Z}={\left({\text{z}}_{i}^{T}\right)}_{i=1}^{n}$; we are here including the intercepts in β and α. The joint posterior *p*(β, α|**y**, **X**, **Z**) for the Rician and NC-χ regression models is intractable, and we instead simulate from the joint posterior using an efficient MCMC algorithm described in section 3.4.

### 3.2. Prior distribution

Our prior distribution for the Rician and the NC-χ model is from the general class in Villani et al. (2012). Let us for clarity focus on the prior for β_0_ and β in $\ln\mu_{i}=\beta_{0}+{\text{x}}_{i}^{T}\beta$; the prior on α_0_ and α in ϕ is completely analogous. We first discuss the prior on the intercept β_0_. Start by standardizing the variables to have mean zero and unit standard deviation. This makes it reasonable to assume prior independence between β_0_ and β. The intercept is then ln μ at the mean of the original variables. The idea is to let the user specify a prior directly on μ when the variables are at their means, and then back out the implied prior on β_0_. Let μ have a log-normal density with mean m^*^ and variance s^*2^. The induced prior on the intercept is then $\beta_{0}\sim N\left(m,s^{2}\right)$ with $s^{2}=\log\left[{\left(\frac{s^{*}}{m^{*}}\right)}^{2}+1\right]$ and *m* = log(m^*^) − *s*^2^/2.

The prior on β needs some care, since its effect on the response comes through a link function, and μ enters the model partly via a non-linear Bessel function. Following Villani et al. (2012), we let β ~ *N*(0, *c*Σ), where $\Sigma ={\left(X^{T}\hat{D}X\right)}^{-1}$ is the Fisher information for β, *X* is the matrix of variables excluding the intercept, and $\hat{D}$ is the Fisher information for μ conditional on ϕ, evaluated at the prior modes of β_0_ and β, i.e., at the vector (*m*,**0**′)′. Thus $\hat{D}$ depends only on the constant *m*. The conditional Fisher information for $\mu ={\left(\mu_{1},\ldots \mu_{n}\right)}^{\prime }$ is a diagonal matrix with elements

$$-E\left[\frac{\partial^{2}\log p\left(y_{i}|\mu_{i},\phi_{i}\right)}{\partial \mu_{i}^{2}}\right]g^{\prime }{\left(\mu_{i}\right)}^{-2}.$$

Setting *c* = *n* gives a unit information prior, i.e., a weak prior that carries the information equivalent to a single observation from the model.

### 3.3. Variable selection

Our MCMC algorithm can perform Bayesian variable selection among both sets of variables (i.e., **x** and **z**). We make the assumption that the intercepts in ln μ and ln ϕ are always included in the model. Let us again focus on β in the equation for μ. Define the vector with binary indicators $I=\left\{I_{1},\ldots I_{p}\right\}$ such that *I*_*j*_ = 0 means that the *j*th element in β is zero, and that the corresponding variable drops out of the model. Let $I^{c}$ denote the complement of I. Let $\beta_{I}$ denote the subset of regression coefficients selected by I. To allow for variable selection we take the previous prior $\beta_{}\sim N\left(0,c\Sigma \right)$ and condition on the zeros in β dictated by I:

$$\begin{array}{lll} \beta_{I}|I & \sim & N\left[0,c\left(\Sigma_{I,I}-\Sigma_{I,I^{c}}\Sigma_{I^{c},I^{c}}^{-1}\Sigma_{I^{c},I}^{T}\right)\right], \end{array}$$

and $\beta_{I^{c}}|I$ is identically zero. To complete the variable selection prior, we let the elements of I to be a priori independent and Bernoulli distributed, i.e., Pr(*I*_*i*_ = 1) = π, and π is allowed to be different for the variables in μ and ϕ. We choose π = 0.5 for both sets of variables in μ and ϕ. Other priors on I are just as easily handled.

### 3.4. Markov Chain Monte Carlo algorithm

We use the Metropolis-within-Gibbs sampler presented in Villani et al. (2012) and Villani et al. (2009). The algorithm samples iteratively from the set of full conditional posteriors, which in our case here are

1. $\left(\beta ,I_{\beta }\right)|\cdot$
2. $\left(\alpha ,I_{\alpha }\right)|\cdot$.

Note that we sample β and $I_{\beta }$ jointly given the other parameters (indicated by ·). The full conditional posteriors $p\left(\beta ,I_{\beta }|\cdot \right)$ and $p\left(\alpha ,I_{\alpha }|\cdot \right)$ are highly non-standard distributions, but can be efficiently sampled using tailored Metropolis-Hastings (MH) updates. The sampling of the pair $\left(\alpha ,I_{\alpha }\right)$ is analoguous to the sampling of $\left(\beta ,I_{\beta }\right)$, so we will only describe the update of $\left(\beta ,I_{\beta }\right)$. The MH proposal distribution is of the form

(4) $$\begin{array}{lll} J\left(\beta_{p},I_{p}|\beta_{c},I_{c}\right) & = & J_{1}\left(\beta_{p}|I_{p},\beta_{c}\right)J_{2}\left(I_{p}|\beta_{c},I_{c}\right), \end{array}$$

where $\left(\beta_{c},I_{c}\right)$ denotes the current and $\left(\beta_{p},I_{p}\right)$ the proposed posterior draw. Following Villani et al. (2009), we choose *J*_2_ to be a simple proposal of I where a subset of the indicators is randomly selected and a change of the selected indicators is proposed, one variable at a time. Following Villani et al. (2012) and Villani et al. (2009), we use a multivariate-*t* distribution with 10 degrees of freedom for the proposal of β, the *J*_1_ distribution. The thicker tails of the t distribution ensures that the sampler does not get stuck. However, we have verified that also a normal proposal works well in our applications in section 4.5. The multivariate-*t* distribution for the *J*_1_ distribution becomes

$$\beta_{p}|I_{p},\beta_{c}\sim t_{10}\left[\hat{\beta },-{\left(\frac{\partial^{2}\log p(\beta |y)}{\partial \beta \partial \beta^{T}}\right)}^{-1}|{}_{\beta =\hat{\beta }}\right],$$

where $\hat{\beta }$ is the terminal point of a small number of Newton iterations to climb toward the mode of the full conditional $p\left(\beta_{p}|I_{p},\cdot \right)$, and − ${\left(\frac{\partial^{2}\log p(\beta |y)}{\partial \beta \partial \beta^{T}}\right)}^{-1}{|}_{\beta =\hat{\beta }}$ is the negative inverse Hessian of the full conditional posterior evaluated at $\beta =\hat{\beta }$. Note that we are for notational simplicity suppressing the conditioning on the variables **X** and **Z**.

Importantly, the number of Newton iterations can be kept very small (one or two steps is often sufficient), since the iterations always start at β_*c*_, which is typically not far from the mode. To implement the Newton iterations we need to be able to compute the gradient $\frac{\partial \log p\left(y|\beta \right)}{\partial \beta }$ and the Hessian $\frac{\partial^{2}\log p\left(\beta |y\right)}{\partial \beta \partial \beta^{T}}$ efficiently. Villani et al. (2012) document that this can be done very efficiently using the chain rule and compact matrix computations, and details for the NC-χ regression are given in Appendix A in Supplementary Material. In DTI, when the parameter space is restricted to the set of positive definite matrices, these expressions need to be extended, see section 4.2.

## 4. Estimating fractional anisotropy and mean diffusivity in DWI data

### 4.1. Diffusion weighted imaging

While fMRI data are mainly specified by the echo time and the repetition time of the pulse sequence, DWI data also require specification of the *b*-value (Le Bihan et al., 1986). The *b*-value in turn depends on two factors; the strength and the duration of the diffusion gradient. Using a larger *b*-value enables more advanced diffusion models, e.g., through HARDI (Tuch et al., 2002; Descoteaux et al., 2008; Gupta and Awate, 2017), which for example can be used to properly account for multiple fiber orientations in a single voxel. A significant drawback of a higher *b*-value is, however, a lower signal to noise ratio. The main reason for this is that the signal decays exponentially with time, and high *b*-values require longer diffusion gradients. As a consequence, Rician noise models are far more common for DWI than for fMRI, as the Rician distribution is only well approximated by a Gaussian for high SNRs.

### 4.2. The diffusion tensor model

The most common diffusion tensor model states that the signal *S*_*i*_ for measurement *i* can be written as

(5) $$\begin{array}{l} S_{i}=S_{0}\exp\left(-b_{i}g_{i}^{T}\text{D}g_{i}\right), \end{array}$$

where *S*_0_ is the signal in absence of any diffusion gradient, *b*_*i*_ is the *b*-value, $g_{i}={\left(g_{ix},g_{iy},g_{iz}\right)}^{T}$ is the gradient vector and

$$D=\left(\begin{array}{ccc} d_{xx} & d_{xy} & d_{xz}\\ d_{xy} & d_{yy} & d_{yz}\\ d_{xy} & d_{yz} & d_{zz} \end{array}\right)$$

is the diffusion tensor. The single-diffusion tensor model in Equation (5) can be written as a regression model of the form in Equation (3) with (see e.g., Koay, 2011)

(6) $$\begin{array}{l} \ln\mu_{i}=\beta_{0}+{\text{x}}_{i}^{T}\beta , \end{array}$$

where β_0_ = ln *S*_0_, β = (*d*_*xx*_, *d*_*yy*_, *d*_*zz*_, *d*_*xy*_, *d*_*yz*_, *d*_*xz*_) and

$${\text{x}}_{i}^{T}=-\left(b_{i}g_{ix}^{2},b_{i}g_{iy}^{2},b_{i}g_{iz}^{2},2b_{i}g_{ix}g_{iy},2b_{i}g_{iy}g_{iz},2b_{i}g_{ix}g_{iz}\right).$$

For single-coil imaging, the noise around μ_*i*_ is Rician, and cannot be well approximated by a Gaussian model for high *b*-values where the signal-to-noise ratio is low. When data are collected by parallel techniques using *L* coils, the noise is either Rician distributed or NC-χ distributed with *L* degrees of freedom. If the composite signal is a complex weighted sum of the *L* signals, the magnitude of the composite signal is Rician distributed. If the simpler sum of squares approach is used for merging the L signals into a single image, the resulting signal is instead NC-χ distributed (Tristán-Vega et al., 2012; Aja-Fernandez and Vegas-Sanchez-Ferrero, 2016).

Note that since the tensor *D* is positive definite, the parameter space of β in Equation (6) is restricted. We here use the Log-Cholesky representation (Koay, 2011) to impose this restriction, where the diffusion tensor *D* is expressed as

$$D\left(\omega \right)=\Omega^{T}\Omega$$

with

$$\Omega =\left(\begin{array}{ccc} e^{\omega_{1}} & \omega_{4} & \omega_{6}\\ 0 & e^{\omega_{2}} & \omega_{5}\\ 0 & 0 & e^{\omega_{3}} \end{array}\right).$$

In this parametrization the vector of regression coefficients β(ω) is given by

$$\left(e^{2\omega_{1}},\omega_{4}^{2}+e^{2\omega_{2}},\omega_{6}^{2}+\omega_{5}^{2}+e^{2\omega_{3}},\omega_{4}e^{\omega_{1}},\omega_{4}\omega_{6}+\omega_{5}e^{\omega_{2}},\omega_{6}e^{\omega_{1}}\right).$$

Most applications with the diffusion tensor model takes the logarithm of the measurements and estimates β with least squares (see Koay, 2011 for an overview). This estimation method therefore does not respect the log link in the mean. One can also argue that it also implicitly assumes Gaussian noise in the sense that least squares equals the maximum likelihood estimate only when the noise is Gaussian. Moreover, it does not guarantee that the estimated tensor is positive definite. We refer to Koay (2011) for an overview of constrained non-linear least squares alternatives.

We will here take a Bayesian approach with Rician noise, using a proper log link and a parametrization that guarantees that the posterior mass is fully contained within the space of positive definite matrices. Existing Bayesian approaches to DTI assume Gaussian noise and use the random walk Metropolis (RWM) algorithm to simulate from the posterior distribution. RWM is easy to implement, but is well known to explore the posterior distribution very slowly (see section 4.4). The Metropolis-within-Gibbs algorithm with tailored proposals and variable selection to reduce the dimensionality of the parameter space presented in section 3.4 can explore the posterior distribution in a much more efficient manner (Villani et al., 2009, 2012). As a result of the non-linear mapping from ω to β, the gradient of the likelihood is modified to

$$\frac{\partial \ln\;p\left(y|\omega \right)}{\partial \omega }={\left(\text{X}\frac{\partial \beta \left(\omega \right)}{\partial \omega }\right)}^{T}\text{g},$$

where **g** is the gradient vector in Villani et al. (2012) and

$$\frac{\partial \beta (\omega )}{\partial \omega }=\left(\begin{array}{cccccc} 2e^{2\omega_{1}} & 0 & 0 & 0 & 0 & 0\\ 0 & 2e^{2\omega_{2}} & 0 & 2\omega_{4} & 0 & 0\\ 0 & 0 & 2e^{2\omega_{3}} & 0 & 2\omega_{5} & 2\omega_{6}\\ \omega_{4}e^{\omega_{1}} & 0 & 0 & e^{\omega_{1}} & 0 & 0\\ 0 & \omega_{5}e^{\omega_{2}} & 0 & \omega_{6} & e^{\omega_{2}} & \omega_{4}\\ \omega_{6}e^{\omega_{1}} & 0 & 0 & 0 & 0 & e^{\omega_{1}} \end{array}\right).$$

The Hessian can be modified accordingly.

The Fisher information based prior presented in section 3.2 can in principle be used for DTI. We have found however that the numerical stability of our MCMC sampler improves if we use an alternative prior, which we now describe. We assume the priors for the intercepts β_0_ ~ *N*(*m*_β_, *d*) and α_0_ ~ *N*(*m*_α_, *d*), independently of the priors for the unrestricted tensor coefficients ω ~ *N*(0, *cI*) and the variance function parameters α ~ *N*(0, *cI*), where *c* = 100 to induce non-informative priors and *I* is the identity matrix. Note that the prior expected value of 0 for α implies that the variance of the underlying complex-valued signal ϕ is centered on the homoscedastic model a priori. To set the prior mean on the intercepts β_0_ and α_0_, note first that the models for μ and σ^2^ in Equation (1) become β_0_ = ln μ_*i*_ and α_0_ = ln ϕ_*i*_ when *b* = 0. It is therefore common in the DTI literature to separately pre-estimate the mean intercept β_0_ by the logarithm of the mean of measurements *y* when *b* = 0, and then subsequently remove these observations from the dataset. This procedure improves the numerical stability of the estimations. In a similar vein, we set the prior expected values, *m*_β_ and *m*_α_ by taking the logarithm of the mean and variance of *y* when *b* = 0, respectively; the observations with zero *b*-values are then removed from the dataset in the remaining estimation. We have found improved numerical stability in the MCMC algorithm if we allow for a positive, but small, prior variance of *d* = 0.01.

### 4.3. Data

We use the freely available MGH adult diffusion dataset from the Human Connectome Project (HCP) (Essen et al., 2013; Setsompop et al., 2013)^1^. The dataset comprise DWI data collected with several different *b*-values, and the downloaded data have already been corrected for gradient nonlinearities, subject motion and eddy currents (Glasser et al., 2013; Andersson and Sotiropoulos, 2016). The DWI data were collected using a spin-echo EPI sequence and a 64-channel array coil (Setsompop et al., 2013), yielding volumes of 140 x 140 x 96 voxels with an isotropic voxel size of 1.5 mm. The data collection was divided into 5 runs, giving data with four different *b*-values: 1,000, 3,000, 5,000, and 10,000 s/mm^2^. The number of gradient directions was 64 for *b* = 1,000 and 3,000 s/mm^2^, 128 for *b* = 5,000 s/mm^2^, and 256 for *b* = 10,000 s/mm^2^. Merging the measurements from the 64 channels into a single image was performed using a complex weighted combination (Setsompop et al., 2013), instead of the more simple sum of squares approach. This is an important fact, as the weighted approach for this data leads to noise with a Rician distribution, instead of the NC-χ distribution resulting from the sum of squares approach (Aja-Fernandez and Vegas-Sanchez-Ferrero, 2016). Prior to any statistical analysis, the function FAST (Zhang et al., 2001) in FSL was used to generate a mask of white brain matter, gray brain matter and cerebrospinal fluid (CSF), to avoid running the analysis on voxels in CSF.

### 4.4. Comparing the Rician and Gaussian models for DTI

We compare the Rician and Gaussian DTI models for the voxels in slice 50 in the middle of the brain. See Wegmann et al. (2017) for a comparison of the Gaussian DTI model with least squares and nonlinear least squares estimates on the same data. We mainly compare the estimation results between the models using the whole dataset with all *b*-values up to *b* = 10,000 s/mm^2^, but also present some results for subsets of the whole dataset with *b*-values up to *b* = 3,000 and 5,000 s/mm^2^, respectively.

The estimated single-diffusion tensors are compared across voxels for the Rician DTI model in Equation (1) to the Gaussian counterpart, with respect to the DTI scalar measures mean diffusivity (MD) and fractional anisotropy (FA). The DTI scalar measures are functions of the eigenvalues λ_1_ ≥ λ_2_ ≥ λ_3_ of the single-diffusion tensor, defined as

$$\begin{array}{l} MD=\frac{\lambda_{1}+\lambda_{2}+\lambda_{3}}{3},FA=\sqrt{\frac{3}{2}}\sqrt{\frac{\overset{3}{\underset{i=1}{\sum }}{\left(\lambda_{i}-MD\right)}^{2}}{\overset{3}{\underset{i=1}{\sum }}\lambda_{i}^{2}}}. \end{array}$$

**Figure 1:**
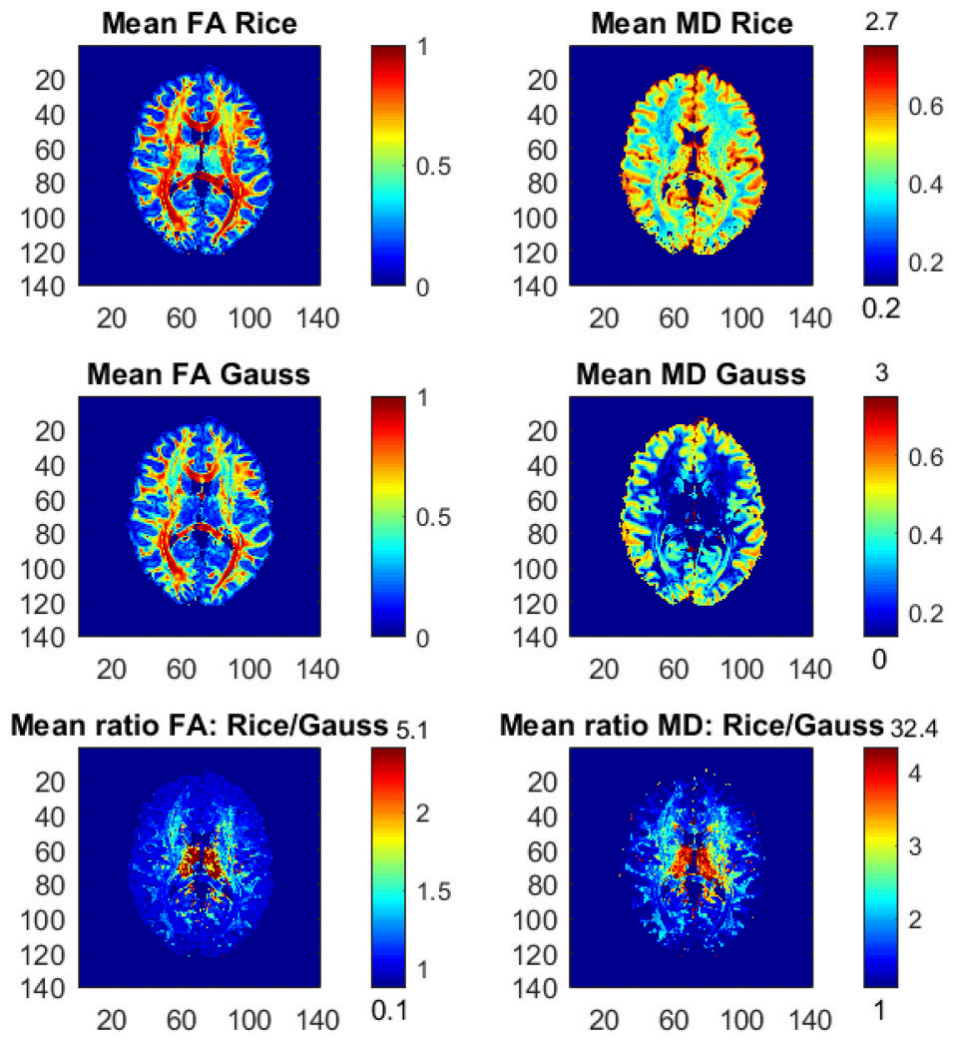
Posterior means and ratios of posterior means of FA and MD for the Rician and Gaussian DTI models, using the whole dataset with all *b*-values up to *b* = 10,000 s/mm^2^. The color bars are shown for the mid 95% values and the minimum and maximum values are marked out at the bottom and top of the color bars, respectively.

**Figure 2:**
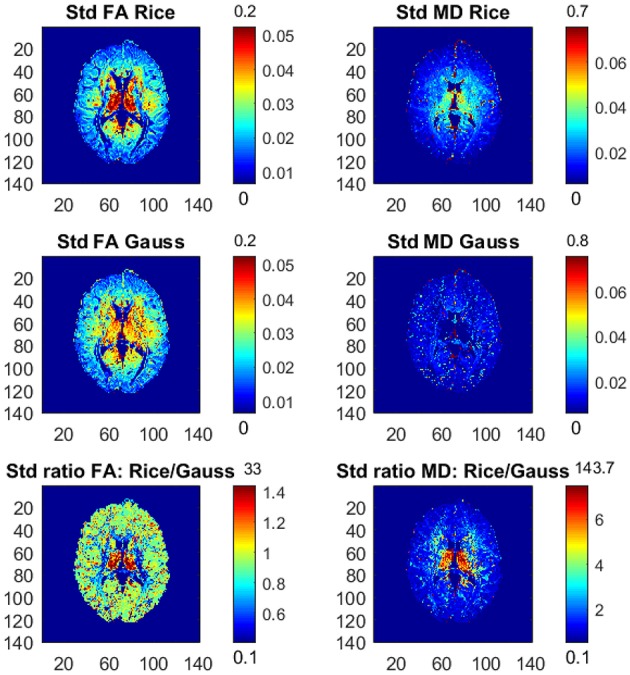
Posterior standard deviations and ratios of posterior standard deviations of FA and MD for the Rician and Gaussian DTI models, using the whole dataset with all *b*-values up to *b* = 10,000 s/mm^2^. The color bars are shown for the mid 95% values and the minimum and maximum values are marked out at the bottom and top of the color bars, respectively.

Figure 1 shows the posterior means of FA and MD and the ratios of posterior means between the models, and Figure 2 shows the posterior standard deviations of FA and MD and the ratios of posterior standard deviations between the models.

In general, the Gaussian model substantially underestimates mean values of FA in many voxels, especially in mid-regions with low or mid-size values of FA, compared to the theoretically correct Rician model. In addition, the Gaussian model greatly underestimates MD across the whole slice of the brain compared to the Rician model. Hence, using the Gaussian model for DTI can therefore lead to severely misleading inferences. The standard deviations of FA and MD are small for both models. In white-matter regions with high FA values the Gaussian model estimates slightly larger standard deviations of FA compared to mid-regions with slightly larger standard deviations of FA for the Rician model. On the other hand, the standard deviations of MD are underestimated by the Gaussian model in all voxels.

**Figure 3:**
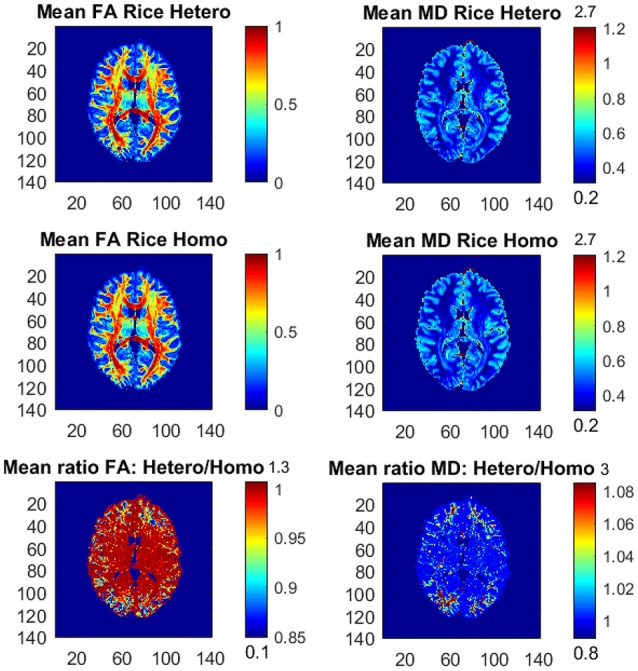
Posterior means and ratios of posterior means of FA and MD for the heteroscedastic (Hetero) and homoscedastic (Homo) Rician DTI models, using the whole dataset with all *b-*values up to *b* = 10,000 s/mm^2^. The color bars are shown for the mid 95% values and the minimum and maximum values are marked out at the bottom and top of the color bars, respectively.

Figure 3 shows the posterior means of FA and MD and the ratios of posterior means for the Rician models with variables in the noise variance ϕ (heteroscedastic model) and without variables in ϕ (homoscedastic model). The differences between the models are small, but in the outer parts of the brain the homoscedastic Rician model slightly overestimates the posterior means of FA in a large number of voxels. The posterior standard deviations of FA and MD for the Rician models are similar, but the homoscedastic Rician model slightly underestimates, in general, the standard deviation of FA in the outer parts of the brain (not shown here). The differences in FA between the Rician models agree with our previous findings that the diffusion variables (directions) especially affect the noise variance for the Rician model in the outer parts of the brain, where directional DTI measures such as FA are affected. This is in contrast to the non-directional measure MD, for which the differences between the models are negligible. Hence, in voxels with heteroscedastic noise variance that depends on the diffusion directions the posterior means and standard deviations of FA are slightly different for the heteroscedastic and homoscedastic Rician models.

**Figure 4:**
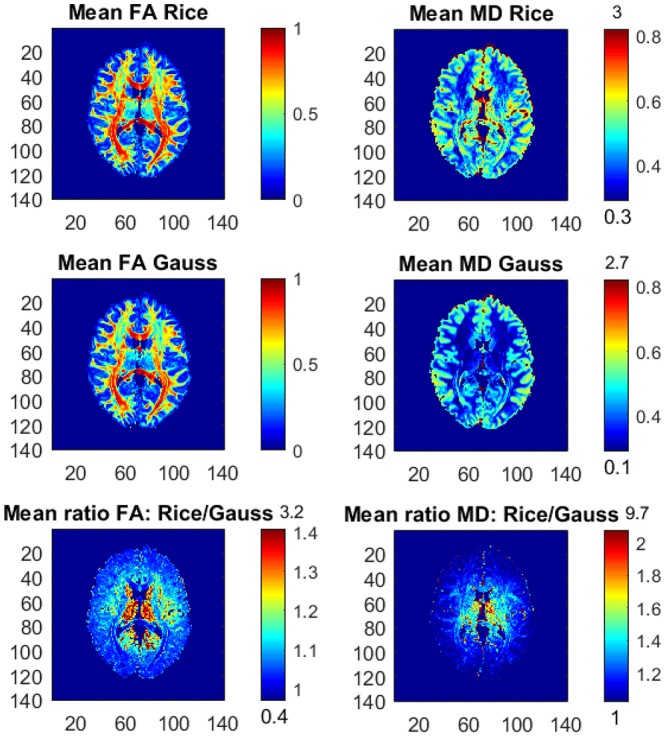
Posterior means and ratios of posterior means of FA and MD for the Rician and Gaussian DTI models, using the part of the dataset with all *b*-values up to *b* = 5,000 s/mm^2^. The color bars are shown for the mid 95% values and the minimum and maximum values are marked out at the bottom and top of the color bars, respectively.

It is relatively uncommon with measurements at a *b*-value of 10,000. Figure 4 therefore shows the posterior means and the ratios of posterior means of FA and MD between the models for the part of the whole dataset with all *b*-values up to *b* = 5,000 s/mm^2^, hence excluding the observations with the highest *b*-value. The differences in FA and MD are notably smaller compared to the results from the whole dataset, but the Gaussian model still underestimates the posterior mean values of FA and MD substantially in many voxels. Taking an even smaller data subset with all *b*-values up to *b* = 3,000 s/mm^2^, the differences in FA and MD between the models become negligible (not shown here).

**Figure 5:**
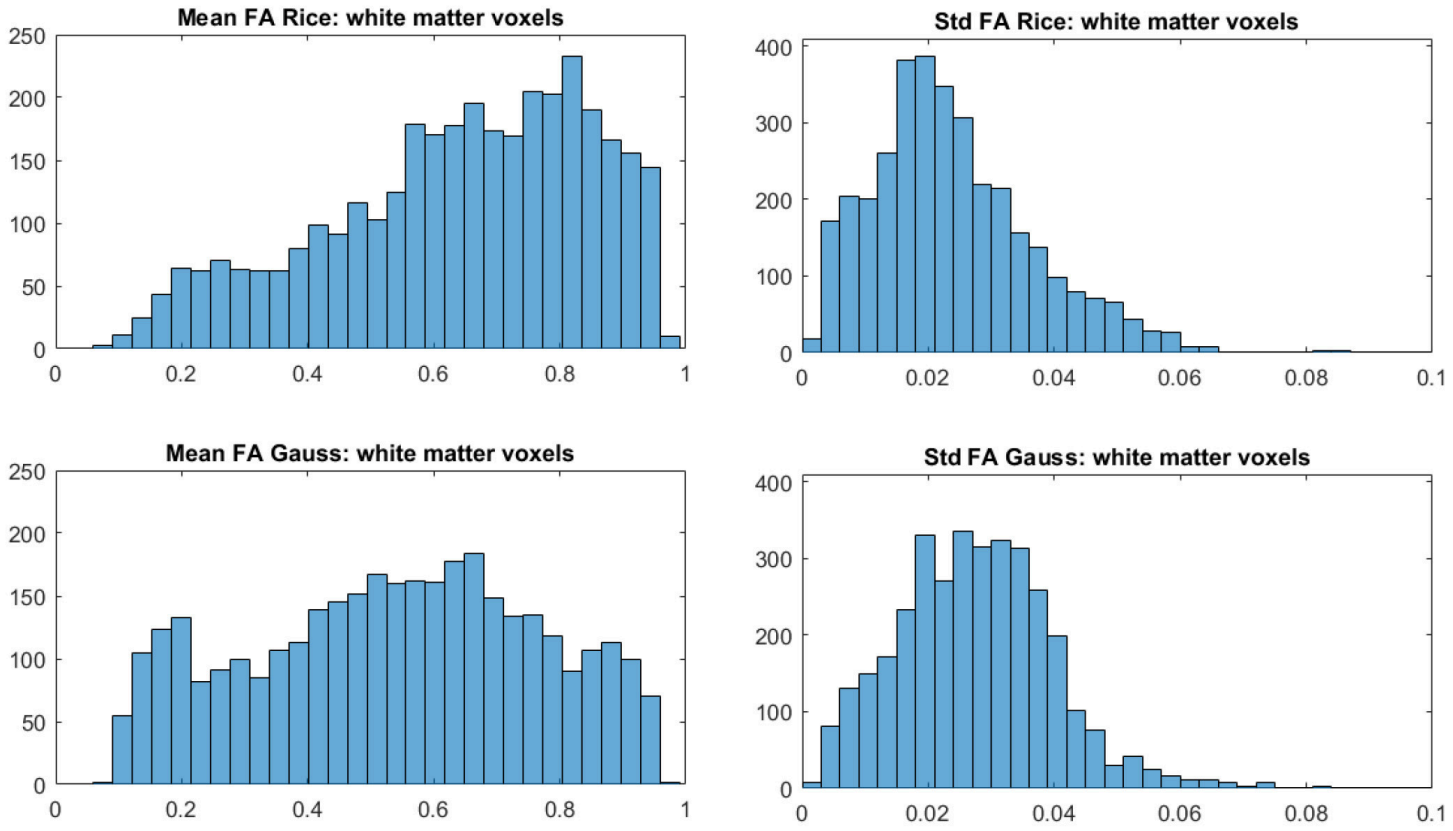
Histograms of posterior means **(left)** and posterior standard deviations **(right)** of FA for white-matter voxels for the Rician and Gaussian DTI models, using the whole dataset. A white-matter voxel is defined as a voxel where the probability is 1 for white matter from the function FAST in FSL.

To investigate the differences between the Rician and Gaussian models in white matter, we use the function FAST in FSL to compute the probabilities for white matter in each voxel of the brain. Let a white-matter voxel be defined as a voxel where the probability is 1 for white matter. It is generally expected that white-matter voxels have high FA. Figure 5 shows that this is true for the Rician model as the distribution of the posterior means of FA is more skewed to larger values, compared to more uniformly distributed posterior means of FA for the Gaussian model. Hence, the Gaussian model underestimates, on average, FA in white-matter voxels.

**Figure 6:**
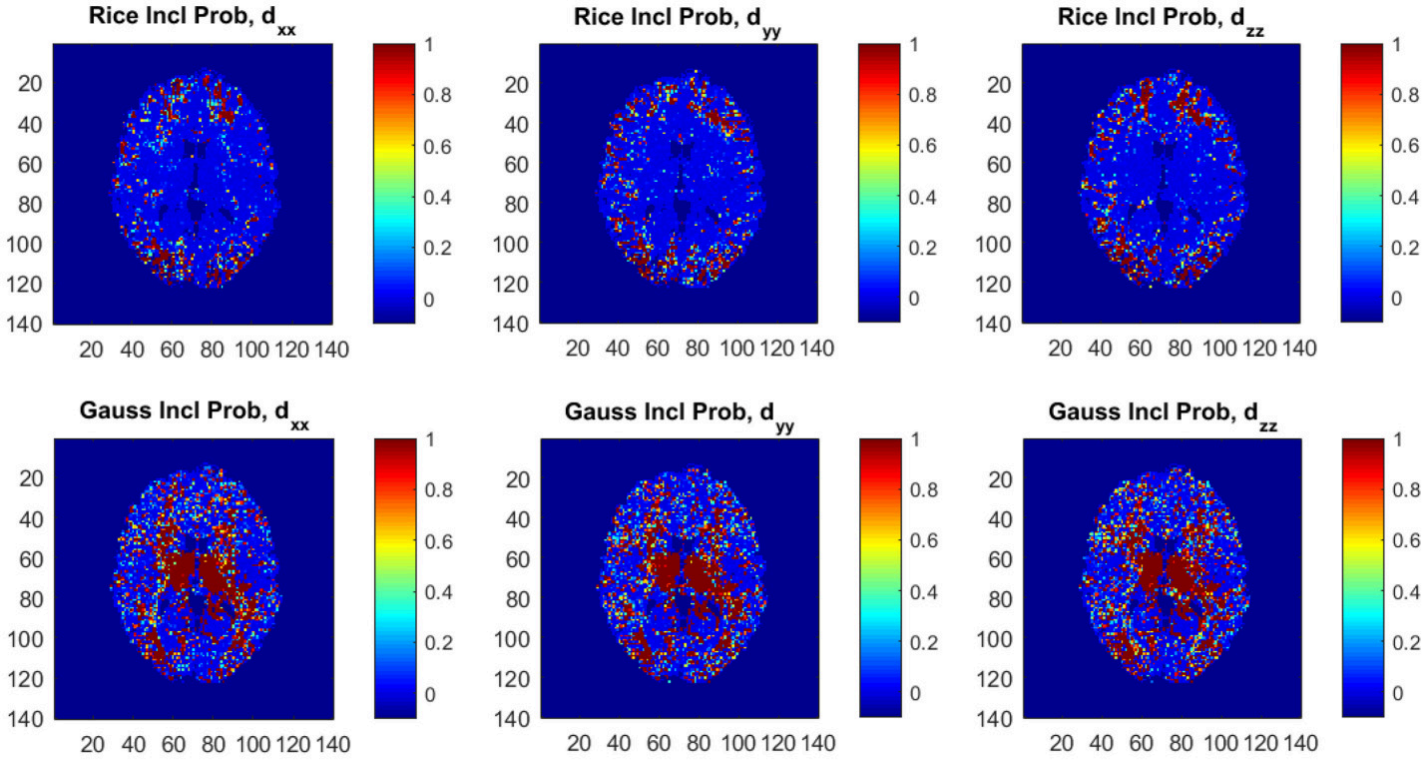
Posterior inclusion probabilities for the variables corresponding to the diffusion directions (*d*_*xx*_, *d*_*yy*_, *d*_*zz*_) in the variance function ϕ for the Rician and Gaussian DTI models. Note that the inclusion probability is close to 1 for a large number of voxels, especially for the Gaussian model.

Figure 6 shows posterior inclusion probabilities for the variables corresponding to (*d*_*xx*_, *d*_*yy*_, *d*_*zz*_) in **z** (the variance function) for both models. In a large number of voxels the inclusion probabilities for the Gaussian model are close or equal to 1, compared to far fewer voxels for the Rician model. This clearly shows that diffusion variables affect the noise variance in both models, and may imply that homoscedastic DTI models can give distorted results as we documented in Wegmann et al. (2017) for the Gaussian DTI model. Using a part of the dataset with all *b*-values up to *b* = 5,000 s/mm^2^ implies far fewer voxels with inclusion probabilities close or equal to 1 for both models, but there are still substantially more voxels with this property for the Gaussian model compared to the Rician model (not shown here).

### 4.5. MCMC convergence and efficiency

The MCMC convergence is excellent for both the Rician and Gaussian DTI models, with high acceptance probabilities for μ and ϕ in almost all voxels for all estimated datasets. The mean MH acceptance probabilities for μ and ϕ are 74 and 87% for the Rician model, compared to 70 and 90% for the Gaussian model. The standard deviations of the acceptance probabilities across voxels are 7.5 and 16.2% for the Rician model, compared to 5.1 and 5.2% for the Gaussian model.

**Figure 7:**
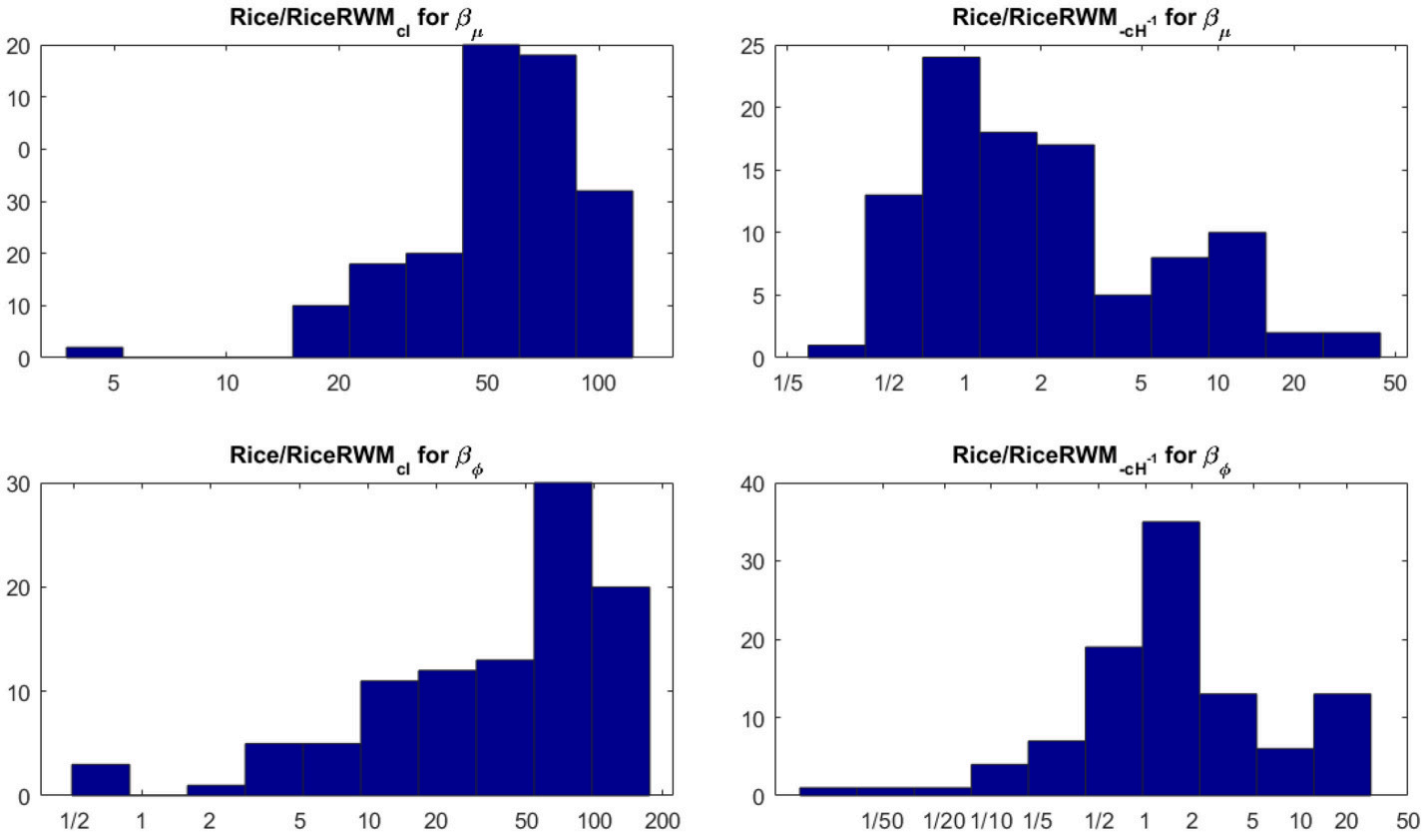
Histograms of the ratio of independent draws per minute for our MCMC algorithm compared to each type of RWM algorithm for 100 randomly sampled white matter voxels for the Rician model. The number of independent MCMC draws is defined as the number of total MCMC draws divided by the estimated inefficiency factor $IF=1+2\overset{\infty }{\underset{k=1}{\sum }}\rho_{k},$ where ρ_*k*_ is the autocorrelation function of lag *k* of the MCMC chain. The rows correspond to the parameters, the columns to the two covariance matrices in the RWM algorithm.

We compare the efficiency of our MCMC algorithm to commonly used Random Walk Metropolis (RWM) algorithms for MCMC in DTI [see e.g., Zhou (2011) and the highly influential work in Behrens et al. (2003)]. The RWM algorithms use a multivariate normal distribution centered on the current parameter value to propose a posterior draw of all parameters in μ and σ in a single block. The most common choice of proposal covariance matrix in DTI is a scaled identity matrix where the scale is chosen adaptively to achieve optimal performance. We also compare our MCMC algorithm to a refined version with covariance matrix −*cH*^−1^, where *H* is the Hessian at the posterior mode and *c* is a scalar which is again chosen adaptively for optimal performance. Figure 7 shows that our MCMC algorithm is much more efficient in almost all voxels than the RWM algorithm with covariance matrix *cI*, and also more efficient than the RWM algorithm with covariance matrix −*cH*^−1^ in most voxels.

## 5. Discussion

We propose a Bayesian non-central χ regression model for neuroimaging with the Rician model as a prominent special case. We use simulated Rician regression data to demonstrate that the Rician model is remarkably adept at recovering the signal even at very low SNRs; we also document that the Gaussian model fails to detect the signal for low SNRs.

Real diffusion data from the Human Connectome Project (Essen et al., 2013) is used to show that the results from the correct Rician DTI model can differ substantially from the approximate Gaussian model typically used for diffusion tensor estimation. The Gaussian model greatly underestimates the mean diffusivity (MD) and substantially underestimates the FA of the single-diffusion tensors, which is consistent with previous results (Andersson, 2008). We also show that the differences between the Rician and Gaussian models increase with the *b*-value, which is natural since the SNR decreases with a higher *b*-value.

Contrary to previous Bayesian and EM approaches, our Bayesian methods work directly on NC-χ or Rician distributions, without the need to introduce missing phase data. Our framework is also more general compared to the work in Andersson (2008) and other approaches, as it is possible to include variables for both the mean and the variance of the noise, and not only variables for the mean. We demonstrate that DTI noise of the underlying complex-valued signal is heteroscedastic, especially for the Gaussian model. This is consistent to our recent work in Wegmann et al. (2017), where we documented that using diffusion variables for the noise variance gives rather different results for DTI. It is also possible to include head motion parameters, and their temporal derivatives, as variables for the noise variance for both fMRI and DTI. This can for example be used to down-weight measurements close to motion spikes (Elhabian et al., 2014; Power et al., 2014; Siegel et al., 2014) (as any measurement with a high variance is automatically down-weighted in our framework). For models with a large number of variables, our variable selection can discard variables of no interest.

A potential drawback of our approach is the computational complexity. It takes 5.6 s to run 1,000 MCMC iterations for the Gaussian model in a representative voxel for the DTI data, and 11.2 s for the Rician model. The processing time was measured using a computer with an Intel Core i7-4790K CPU, 4 physical cores and 32 GB of memory. For a typical DTI dataset with 20,000 brain voxels, this gives a total processing time of 7.8 h for the Gaussian model and 15.6 h for the Rician model. For this reason, we have only analyzed a single subject, as a group analysis with 20 subjects would be rather time consuming. As each voxel is analyzed independently, it is in theory straightforward to run MCMC on the voxels with a highly parallel structure, using a CPU or a GPU (Guo, 2012; Eklund et al., 2013).

We have focused on the rather simple single-diffusion tensor, while more recent work focus on extending the diffusion tensor to higher orders. In Westin et al. (2016), a regression approach is used to estimate the diffusion tensor and a fourth order covariance matrix in every voxel. Our regression framework can therefore easily be applied to QTI (q-space trajectory imaging) data (Westin et al., 2016) as well, and more generally for any diffusion model that can be estimated using regression. As a fourth order covariance matrix contains 21 independent variables, the possibility to perform variable selection becomes even more important. Furthermore, DTI is still the most common choice for studies investigating FA differences between healthy controls and subjects with some disease (Shenton et al., 2012; Eierud et al., 2014). Another indicator of the importance of FA is that the TBSS approach (Smith et al., 2006) has received more than 3,541 citations (with 531 citations in 2016). Our approach gives the full posterior distribution of the FA, and any other function of the diffusion tensor, which can be used for tractography and to down-weight subjects with a higher uncertainty in a group analysis. This is in contrast to TBSS and the work in Andersson (2008), which ignore the uncertainty of the FA. Andersson (2008) develops a sophisticated maximum a posteriori (MAP) estimation for the DTI model, but does not deal with posterior uncertainty, in contrast to our full MCMC sampling from the posterior distribution.

## Author contributions

All authors listed have made a substantial, direct and intellectual contribution to the work, and approved it for publication.

### Conflict of interest statement

The authors declare that the research was conducted in the absence of any commercial or financial relationships that could be construed as a potential conflict of interest.

## Footnotes

^1^http://www.humanconnectome.org/documentation/MGH-diffusion/
