# Supplementary material for: Bayesian Rician Regression for Neuroimaging
Source: Front Neurosci. 2017 Oct 20;11:586. doi: 10.3389/fnins.2017.00586 (PMC5655010; doi:10.3389/fnins.2017.00586)
Supplement: Supplementary file 1 [file Presentation1.pdf]

Appendix A: Gradients and Hessians

Villani *et al.* [1] derive the gradient and the Hessian for a general posterior of the form

$$p(\beta|\mathbf{y}, \mathbf{x}) \propto \prod_{i=1}^{n} p(y_i|\phi_i, \mathbf{x}_i)p(\beta), \quad (1)$$

where $k(\phi_i) = \mathbf{x}_i'\beta$ is a smooth link function, and $\mathbf{x}_i$ is a variable vector for the ith observation; the full conditional posteriors for β and α in the Rician and the NC-χ case with logarithmic links are clearly of this form.

The general expressions of the gradient and Hessian in [1] for the model class in Equation (3) show that it is sufficient for our MCMC algorithm to compute the first and second derivatives with respect to (w.r.t.) μ and ϕ, respectively, for each of the individual observation. The log-likelihood for one observation y of a non-central χ variable can be written as [2]

$$\ln p(y|\mu, \phi, L) = L \ln y - \ln \phi - (L-1)\ln \mu - \frac{(y^2+\mu^2)}{2\phi} + \ln I_{L-1}\left(\frac{y\mu}{\phi}\right),$$

where $I_{L-1}(\cdot)$ is the modified Bessel function of the first kind of order $L-1$. Let $z = \frac{y\mu}{\phi}$ and

$$B(z) = \frac{I_1(z)}{I_0(z)}, \text{ if } L = 1$$

$$B(z) = \frac{I_{L-2}(z) + I_L(z)}{2I_{L-1}(z)}, \text{ if } L \geq 2$$

Then,

$$B'(z) = \frac{dB(z)}{dz} = \frac{1}{2}\left(1 + \frac{I_2(z)}{I_0(z)}\right) - B^2(z), \text{ if } L = 1$$

$$B'(z) = \frac{dB(z)}{dz} = \frac{1}{4}\left(3 + \frac{I_3(z)}{I_1(z)}\right) - B^2(z), \text{ if } L = 2$$

$$B'(z) = \frac{dB(z)}{dz} = \frac{1}{4}\left(2 + \frac{I_{L-3}(z) + I_{L+1}(z)}{I_{L-1}(z)}\right) - B^2(z), \text{ if } L \geq 3.$$

1) Derivatives w.r.t. to μ::

$$\frac{\partial \ln p(y|\mu, \phi, L)}{\partial \mu} = \frac{yB(z) - \mu}{\phi} - \frac{L-1}{\mu}$$

$$\frac{\partial^2 \ln p(y|\mu, \phi, L)}{\partial \mu^2} = \left(\frac{y}{\phi}\right)^2 B'(z) - \frac{1}{\phi} + \frac{L-1}{\mu^2}$$

2) Derivatives w.r.t. to ϕ::

$$\frac{\partial \ln p(y|\mu, \phi, L)}{\partial \phi} = \frac{1}{2}\left(\frac{y^2+\mu^2}{\phi^2}\right) - \frac{1}{\phi}(1 + zB(z))$$

$$\frac{\partial^2 \ln p(y|\mu, \phi, L)}{\partial \phi^2} = \frac{z[B(z) + zB'(z)] - \frac{1}{2}\left(\frac{y^2+\mu^2}{\phi}\right)}{\phi^2} - \frac{\partial \ln p(y|\mu, \phi, L)}{\partial \phi}\phi^{-1}$$

Appendix B: Signal detection capability - a simulation study

We will here investigate the capability of the Rician and Gaussian regression models to accurately detect the signal in Rician data. Note that the opposite experiment of simulating Gaussian data and assessing the performance of the Rician model is not possible since the negative values obtainable under the Gaussian have zero probability in the Rician distribution. Since many models for fMRI, DWI and QTI data can all be written as Rician regression models, we are here interested in assessing the performance on the general Rician *regression* model, regardless of its specific application in neuroimaging. We have nevertheless chosen to present the results as if the data came from an fMRI experiment. The reason for this is that fMRI data are familiar to many readers, and it also makes it possible to illustrate signal detection visually as activity localization in brain images. We could alternatively have visualized the results as diffusion data, but this is more complex and would require a DWI simulator.

The data are simulated from a model that mimic the results from a real fMRI experiment with a simple block paradigm. The real fMRI data had a spatial resolution of 1.6 x 1.6 x 1.8 mm^3, and the noise variance and the variance for the activation parameter was manipulated in the simulated datasets to obtain a pre-specified level of activation and SNR. The noise variance experimentally controls the SNR levels, while the variance for the activation parameter is adjusted to accommodate one of four chosen t-ratios (0, 3, 5 and 7) for each of the voxels on our selected slice of the brain.

The prior distributions on the parameters in the Rician and Gaussian model are carefully chosen to carry the same information in both models. Specifically, we choose unit information priors (see Section 3.2), such that the priors only carry the information from a single observation in each of the models.

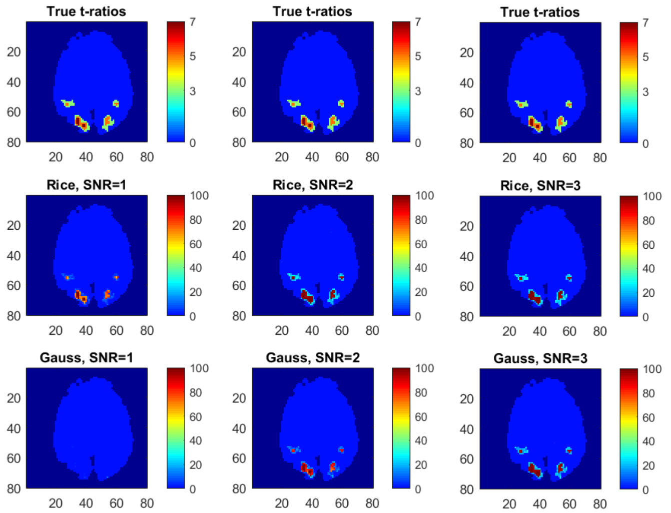

Fig. 1. Comparison of activation inferences using the Rician and Gaussian models on simulated fMRI data. Top row: True activations in the Rician data generating model. Middle (Rice) and bottom (Gauss) row: Percentage of simulated datasets where the posterior probability of activation, $\Pr(\beta_j > 0|\mathbf{y}, \mathbf{X}, \mathbf{Z})$, is larger than 99 %. Color should be used in print.

We simulate 100 datasets for each SNR level. The first row with graphs in Figure 1 shows the four activated regions in the data generating process in the form of "t-ratios" (parameter value/standard deviation). The second row of graphs show that all four activation regions are correctly localized with our Rician model in a large majority of the simulated datasets, while the third row shows that the Gaussian model completely misses all of the activated regions when SNR=1, and has a high failure rate when SNR=2.

References

[1] Mattias Villani, Robert Kohn, and David Nott, "Generalized smooth finite mixtures," *Journal of Econometrics*, vol. 171, no. 2, pp. 121–133, 2012.

[2] Antonio Tristán-Vega, Santiago Aja-Fernández, and Carl-Fredrik Westin, "Least squares for diffusion tensor estimation revisited: Propagation of uncertainty with rician and non-rician signals," *NeuroImage*, vol. 59, no. 4, pp. 4032–4043, 2012.
